# Maintained activity in ankylosing spondylitis patients treated with TNFi and/or NSAID for at least 12 weeks: a cross-sectional study in Brazil

**DOI:** 10.1186/s42358-022-00270-3

**Published:** 2022-10-28

**Authors:** Ricardo Acayaba de Toledo, Felipe Merchan Ferraz Grizzo, Vander Fernandes, Renato Calheiros, Ricardo T. Russo, Gustavo Rosal, Luiz Roberto Delboni Marchese, Roberto Tunala, Renato Watanabe, Marina Gabriela Birck, Guilherme Silva Julian, Francisco Jose Forestiero

**Affiliations:** 1 Fundação Faculdade Regional de Medicina de São José do Rio Preto, São José do Rio Preto, SP Brazil; 2 Paraná Medical Research Center, Maringá, PR Brazil; 3 Centro De Infusão de Biológicos - CIB, Cuiabá, MT Brazil; 4 Novartis Pharmaceuticals Corporation, East Hanover, NJ USA; 5 Novartis Biociências S.A, 90 São Paulo, 04636-000 São Paulo, SP Brazil; 6 IQVIA Brasil, São Paulo, SP Brazil

**Keywords:** Ankylosing spondylitis, Axial radiographic spondyloarthritis, Disease activity, Nonsteroidal anti-inflammatory drug, Patient-reported outcome, Real-world study, Tumor necrosis factor inhibitors

## Abstract

**Background:**

The aim of this study was to evaluate disease activity among patients with axial spondyloarthritis (AS) treated with tumor necrosis factor inhibitors (TNFi) and/or nonsteroidal anti-inflammatory drugs (NSAIDs) for at least 12 weeks in private outpatient settings in Brazil.

**Methods:**

This was a cross-sectional, real-world study conducted in 17 Brazilian private health care institutes. Patients were selected if diagnosed with AS or axial radiographic spondyloarthritis (AxSpA) and treated with NSAIDs or TNFi for at least 12 weeks within the last 26 weeks prior to enrollment. The data were collected from interviewed-based and self-administered questionnaires from patients and physicians. Disease activity was defined as active (≥ 4), low /suboptimal (≥ 2 and < 4) and inactive (< 4) by Bath AS Disease Activity Index (BASDAI) and/or very high (≥ 3.5), high (≥ 2.1 to < 3.5), low (≥ 1.3 to < 2.1), and inactive (< 1.3) by AS Disease Activity Score (ASDAS-CRP). Both patients and physicians’ perceptions of disease control were assessed using a numeric rating scale (NRS; 0—inactive to 10—very active disease).

**Results:**

The cohort included 378 patients with a mean age of 46 years, and the median time since diagnosis until enrollment was 5.4 years (interquartile range 2.7–10.5). Most patients were treated with TNFi alone (74%), followed by TNFi in combination with NSAID (15%), and NSAID alone (11%). About half AS patients showed active disease and 24% of patients showed low activity/suboptimal disease control despite having been treated for at least 12 weeks. Although TNFi showed better disease control than NSAID, inactive disease was experienced by few patients. The NRS (mean [standard deviation]) score for disease perception was 4.24 (3.3) and 2.85 (2.6) for patients and physicians, respectively.

**Conclusion:**

This real-world study showed that most AS patients on TNFi and/or NSAID had not achieved an adequate disease control, as almost 75% of them exhibited active disease or low activity/suboptimal disease control. There remains a need for improved disease management among patients with AS.

**Supplementary Information:**

The online version contains supplementary material available at 10.1186/s42358-022-00270-3.

## Background

Ankylosing spondylitis (AS) is a chronic inflammatory disease that primarily affects the axial joints [1]. It is estimated to affect 0.02 to 0.8% of the Latin American population [2, 3]. AS is characterized by an insidious onset of inflammatory low back pain, with or without peripheral arthritis or extra-articular manifestations [4]. AS is not only associated with a significant clinical and economic burden [5] but with impaired quality of life as well.

National and international guidelines for AS recommend nonsteroidal anti-inflammatory drugs (NSAIDs) as the first-line of pharmacological treatment of AS [6–8]. Biological disease-modifying antirheumatic drugs, such as tumor necrosis factor inhibitors (TNFi) and interleukin-17 (IL-17) inhibitor, are recommended for patients with high disease activity with AS after at least 2 NSAIDs, with current practice starting with TNFi [6, 7, 9].

Monitoring of disease activity, function, mobility, and radiographic progression is highly recommended to investigate whether treatments are leading to complete clinical remission or low disease activity [6, 8]. The disease monitoring includes measuring disease activity by using composite indices for disease activity (Ankylosing Spondylitis Disease Activity Score [ASDAS] or Bath Ankylosing Spondylitis Disease Activity Index [BASDAI]) and laboratory tests (C-reactive protein [CRP]) and imaging, and patient-reported outcomes (PRO) capturing patient perspectives [6, 7].

Over the last decade, the management of AS has changed dramatically. However, a few clinical trials also showed that not all patients could achieve complete clinical remission or adequate disease control [10–12]. Similarly, a few observational studies also showed high disease activity and low activity/suboptimal control following treatment in patients with AS especially in middle-income countries like Brazil [13, 14]. However, limited data are available on disease activity among patients with AS in real-world settings.

Therefore, this cross-sectional study, INVISIBLE-BRAZIL (Making the INVISIBLE visible), aimed to evaluate disease activity among AS patients treated with TNFi and/or NSAID for at least 12 weeks in Brazilian private health care institutes.

## Methods

### Study design and participants

The INVISIBLE-BRAZIL study was a multi-center, observational, cross-sectional, noninterventional study conducted among patients with AS treated with tumor necrosis factor inhibitors (TNFi) and/or NSAIDs. This study was conducted in 17 Brazilian private health care institutes from June 2019 to June 2020.

Eligibility criteria included patients with a diagnosis of AS or axial radiographic spondyloarthritis (AxSpA) according to physician evaluation (modified New York criteria or ASAS classification criteria were not mandatory), aged ≥ 18 years old, treated with at least one TNFi and/or NSAID for at least 12 weeks in the last 26 weeks prior to study enrollment. Patients on interleukin-17, or those who had any severe concomitant disease that might influence rheumatic disease evaluation such as neoplasia, noncontrolled psychiatric disease were excluded. Additionally, patients who were not able to read, understand, and complete the questionnaires and/or who were participating in any other study including administration of drug or procedure were excluded.

All patients (participants) and site investigator (physician) were asked to complete the PROs questionnaires. Patients were treated with standard of care according to physician’s decision and the treatment was retrospectively assessed.

### Data source

Data were collected by the physician, directly from the patients during the single study visit from interviewed-based and self-administered questionnaires, and from patients’ medical records. Patients and their physicians answered the reported outcomes questionnaires to assess disease activity and their perceptions about the disease. Data unrelated to PROs were retrieved from patients’ medical charts. All data were entered into an electronic case report form (eCRF), which constituted the database.

### Disease activity assessment

Disease activity was assessed by two PROs that are commonly used and recommended by standard guidelines [6, 7]: Bath Ankylosing Spondylitis Disease Activity Index (BASDAI) [15], which was entirely self-reported, and ASDAS-CRP [16], which included an inflammatory marker in addition to the self-reported questions. All patients answered BASDAI, but ASDAS-CRP was only evaluated for those with CRP test results in the 30 days prior to the survey.

The BASDAI Index [6, 17] consists of the assessment of five AS symptoms (fatigue, back pain, peripheral joint involvement, enthesitis points, and stiffness) that are evaluated on a numeric rating scale (NRS) varying from 0 to 10 (one being no problem and 10 being the worst problem). The score is obtained by considering the 2 questions regarding stiffness as a single component (average scores of both), and then the average of the 5 partial scores is calculated. Cut-off used to classify disease activity as active was score ≥ 4, and inactive was < 4; an additional analysis was carried out to assess low activity/suboptimal disease control, using the exploratory threshold value of score ≥ 2 and < 4 determined by the study of the disease and references [12, 18–20].

Similarly, ASDAS-CRP [6, 16, 21] includes questions answered as NRS related to back pain, stiffness, patient global assessment, peripheral pain, and swelling, but combines C-reactive protein (CRP), an objective laboratory measure of inflammation frequently used to monitor AS. Based on this score, disease activity is categorized as very high activity (≥ 3.5), high activity (≥ 2.1 to < 3.5), low activity(≥ 1.3 to < 2.1), and inactive disease (< 1.3).

Furthermore, both patients and physicians’ perceptions of disease control were assessed using an NRS (0—inactive to 10—very active disease): according to patients’ perceptions, how active was their rheumatic disease during the last week and according to physicians’ perceptions, how was the disease activity of the patient at the time of medical visit.

### Statistical analysis

Descriptive analyses were used. Data were described as measures of central tendency (means, medians) and dispersion (standard deviation [SD], interquartile range [IQR]) for continuous variables, and absolute number and percentage for categorical variables. Any missing data was considered as missing information, and no data imputation method was performed.

Chi-squared test was used to compare frequencies, and analysis of variance (ANOVA) was used to compare means of two or more independent groups when continuous variable followed normal distribution. Spearman (ρ) was used for evaluating the correlation between two continuous variables that had no normal data distribution. A Pearson correlation coefficient of 0.2 is considered small effect, 0.5 (medium) and a 0.8 or higher high correlation [22]. A P-value < 0.05 was considered statistically significant. Also, the ability of physicians or patients to predict real control of disease was analyzed graphically using a receiver operating characteristic curve. The area under the curve (AUC) was displayed for each analysis.

The study sample size was based on statistical precision and allowance of an outcome to have sufficient generalizability. Our sample included 378 individuals, which was adequate to achieve a robust estimation of the population mean (95% confidence interval, level of significance 0.05). It was based on an acceptable error of 5%,

a Brazilian population of 200.4 million inhabitants with an AS prevalence of 0.5% varying from 0.08 to 1.4% [23], and assuming that about 50% of AS patients in our study cohort would have had BASDAI < 4 based on literature data [12, 18].

Analysis was done using SAS 9.4 (SAS Institute, Cary NC).

### Ethical committee approval

The study was conducted in accordance with the STROBE (Strengthening the Reporting of Observational Studies in Epidemiology) guidelines and with the ethical principles laid down in the Declaration of Helsinki and local ethical regulation. In addition, the study had ethics committee approval of all participating research centers. All patients provided written informed consent prior to participating in the study.

## Results

### Patients’ characteristics

Overall, 386 patients were screened, of whom 378 (97.9%) were eligible and completed the study; 4 patients were not eligible (they were not treated for at least 12 weeks) and data from 4 patients could not be evaluated during monitoring activities, so they were not included in the study cohort.

Of the enrolled participants, 213 (56.3%) were male; the mean (SD) age was 46.4 (13.1) years, and the majority of them were employed (58.5%), overweighted and obese (70%), and had no smoking history 294 (77.8%). Mean age of symptoms’ onset was around 32.6 (SD 13.5) years, and patients were diagnosed at mean age of 39.2 (SD 13.8) years. The median time from diagnosis to enrollment was 5.4 years (IQR 2.7–10.5). In total, 277 (73.3%) patients were screened for the presence of HLA-B27 antigen, of whom 181 (65.3%) were positive. Moreover, 36.2% (137) of the patients had undergone CRP evaluation in the past 30 days prior to enrollment, 67.9% of patients had CRP levels lower than 1 mg/dL. Similar characteristics were found for treatment groups, though TNFi group had a higher presence of male and employed people, and had longer time from AS diagnosis (Table 1).

**Table 1:** Clinical and demographic characteristic of patients enrolled in INVISIBLE-BRAZIL study

	All patients (N = 378)	Using only NSAID (n = 42)	Using only TNFi (n = 281)	Using both TNFi and NSAID (n = 55)
Male, n (%)	213 (56.35)	17 (40.48)	174 (61.92)	22 (40.0)
Age (years), mean (SD)				
At study enrollment	46.40 (13.10)	45.7 (11.6)	46.0 (13.5)	49.1 (12.0)
At AS symptoms onset	32.57 (13.52)	34.83 (13.35)	32.17 (13.48)	32.92 (13.92)
At AS diagnosis	39.24 (13.75)	40.97 (12.45)	38.35 (13.89)	42.45 (13.63)
BMI (kg/m²), n (%)				
< 18.5	1 (0.26)	0	1 (0.36)	0
18.5 to 24.9	112 (29.63)	19 (45.24)	80 (28.47)	13 (23.64)
25 to 29.9	161 (42.59)	16 (38.10)	120 (42.70)	25 (45.45)
30 to 39.9	99 (26.19)	6 (14.29)	77 (27.40)	16 (29.09)
≥ 40	5 (1.32)	1 (2.38)	3 (1.07)	1 (1.82)
Employment status, n (%)				
Employed	221 (58.47)	19 (45.24)	174 (61.92)	28 (50.91)
Unemployed	18 (4.76)	3 (7.14)	12 (4.27)	3 (5.45)
Retired	82 (21.69)	8 (19.05)	61 (21.71)	13 (23.64)
Medical leave	6 (1.59)	0 (0.00)	4 (1.42)	2 (3.64)
Other	51 (13.49)	12 (28.57)	30 (10.68)	9 (16.36)
Smoking habits, n (%)				
Never smoked	294 (77.78)	31 (73.81)	221 (78.65)	42 (76.36)
Former smoker	61 (16.14)	7 (16.67)	47 (16.73)	7 (12.73)
Current smoker	23 (6.08)	4 (9.52)	13 (4.63)	6 (10.91)
Time since first diagnosis*(years), median (IQR)	5.41 (2.66–10.51)	3.43 (1.09–6.60)	6.02 (3.03–11.01)	3.68 (1.43–9.85)
Genetic test for HLA-B27, n (%)	N = 277	N = 35	N = 201	N = 41
Positive	181 (65.34)	20 (57.14)	134 (66.67)	27 (65.85)
CRP in the past 30 days* (mg/dL), n (%)	N = 137	N = 19	N = 96	N = 22
< 0.1	24 (17.5)	3 (15.79)	18 (18.75)	3 (13,64)
0.1 to 1.0	93 (67.9)	13 (68.42)	66 (68.75)	14 (63.64)
> 1.0	20 (14.6)	3 (15.79)	12 (12.5)	5 (22.73)
Mean (SD)	1.65 (12.21)	1.10 (2.50)	0.52 (0.82)	7.06 (30.35)

*Based on enrollment date (informed consent form signature)

*AS* ankylosing spondylitis, *BMI* body mass index, *HLA-B27* human leukocyte antigen B27, *IQR* interquartile range, *SD* standard deviation

### Treatment characteristics and disease control

Most patients (74% [281/378]) were treated with TNFi alone, whereas 15% (55/378) of patients were taking TNFi in combination with NSAID. Considering patients who used only NSAID (11% [42/378]), five used ≥ 2 NSAIDs in the past 26 weeks prior to study enrollment. (Table 2).

**Table 2:** Disease activity according to treatment

		All patients (n = 378)	Using only NSAID (n = 42)	Using only TNFi (n = 281)	Using both TNFi and NSAID (n = 55)	P-value
**BASDAI score -** n (%)						< 0.01*
Inactive disease (< 2)		103 (27.25)	2 (4.76)	97 (34.52)	4 (7.27)	
Low activity/suboptimal control (≥ 2 and < 4)		90 (23.81)	9 (21.43)	75 (26.69)	6 (10.91)	
Active disease (≥ 4)		185 (48.94)	31 (73.81)	109 (38.79)	45 (81.82)	
**BASDAI mean (SD)**		4.06 (2.67)	6.07 (2.50) ^*b*^	3.43 (2.50) ^*a*^	5.77 (2.09) ^*b*^	< 0.01**
**ASDAS-CRP score -** n (%)						< 0.01*
Inactive disease (< 1.3)		36 (26.28)	1 (5.26)	33 (34.38)	2 (9.09)	
Low activity/suboptimal control (≥ 1.3 - <2.1)		31 (22.63)	2 (10.53)	28 (29.17)	1 (4.55)	
High activity (≥ 2.1 - <3.5)		49 (35.77)	11 (57.89)	25 (26.04)	13 (59.09)	
Very high activity (≥ 3.5)		21 (15.33)	5 (26.32)	10 (10.42)	6 (27.27)	
**ASDAS-CRP mean (SD)**		2.28 (1.18)	3.01 (0.90) ^*b*^	1.96 (1.07) ^*a*^	3.07 (1.24) ^*b*^	< 0.01**

*AS* ankylosing spondylitis, ASDAS-CRP Ankylosing Spondylitis Disease Activity Score, *BASDAI* Bath Ankylosing Spondylitis Disease Activity Index, *NSAID* nonsteroidal anti-inflammatory drug, *SD* standard deviation, *TNFi* tumor necrosis factor inhibitors

The most used TNFi was adalimumab (126, 45%), followed by etanercept (54, 19%) and infliximab (48, 17%); whereas the most commonly used NSAIDs were naproxen and celecoxib, followed by etoricoxib and diclofenac. Supplementary Tables 1 and Supplementary Table 2 detail the treatment characteristics in terms of treatment duration, commonly used dosage and frequency of TNFi and NSAIDs, respectively.

**Fig. 1:**
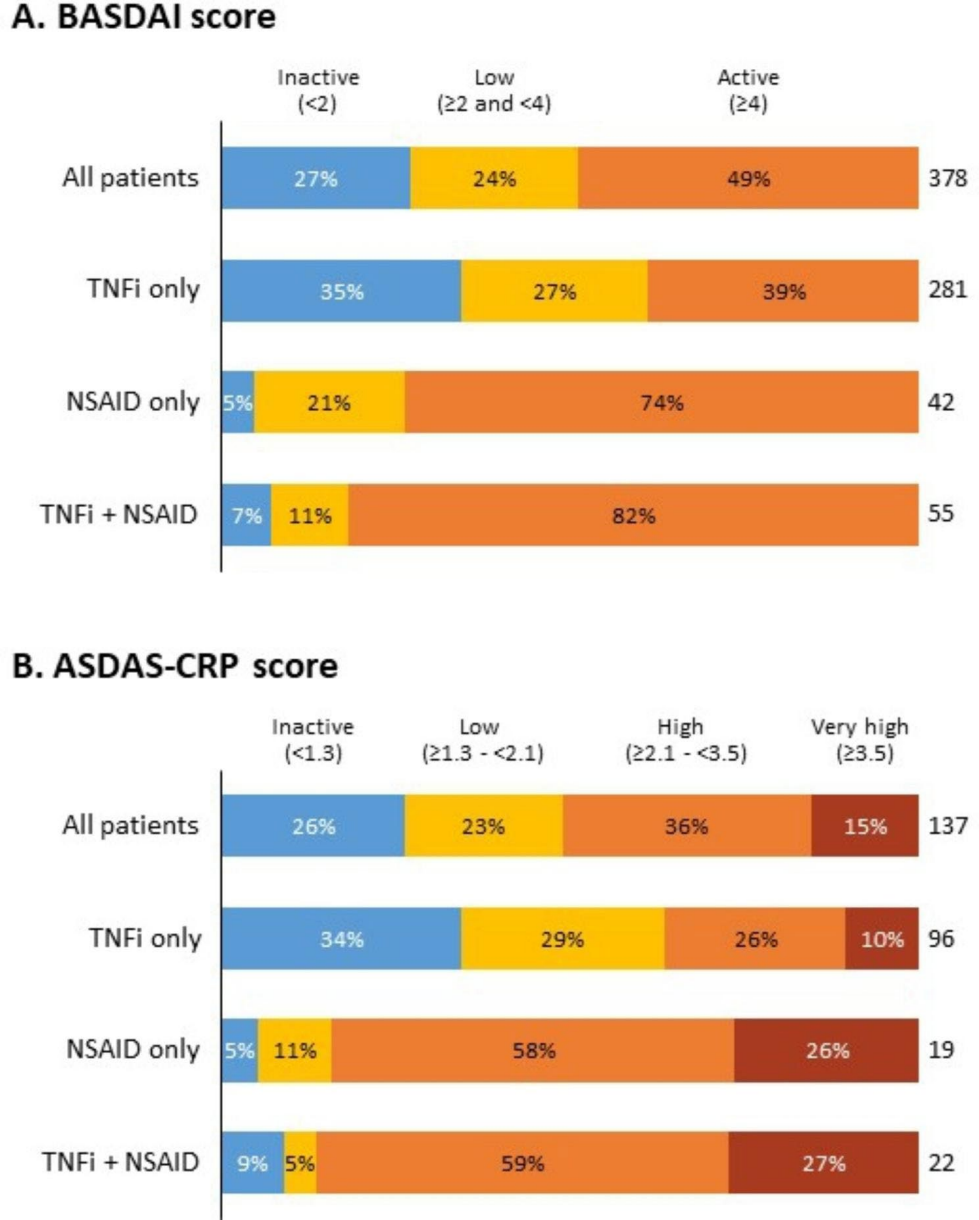
Disease activity according to (A) BASDAI and (B) ASDAS-CRP, as the number and percentage of patients defined as inactive (blue), low disease activity (yellow), active or high activity (orange), and very high activity [ASDAS-CRP only] (dark orange)

Table 4 shows the proportion of patients with active and inactive disease, classified according to BASDAI and/or ASDAS-CRP scores (as illustrated at Fig. 1), and the mean score per treatment type. About half of AS patients had active disease at the time of study enrollment despite having been treated for at least 12 weeks; moreover, only 26–27% had inactive disease, while the others presented with low disease activity/suboptimal disease control. Only about one-third of these patients had inactive and well controlled disease, with those treated with TNFi alone (~ 34% with inactive disease) with better disease control than those treated with TNFi in combination with NSAID (~ 7–9%) or NSAID alone (~ 5%); >73% of patients on TNFi in combination with NSAID or NSAID alone had active disease.

**Fig. 2:**
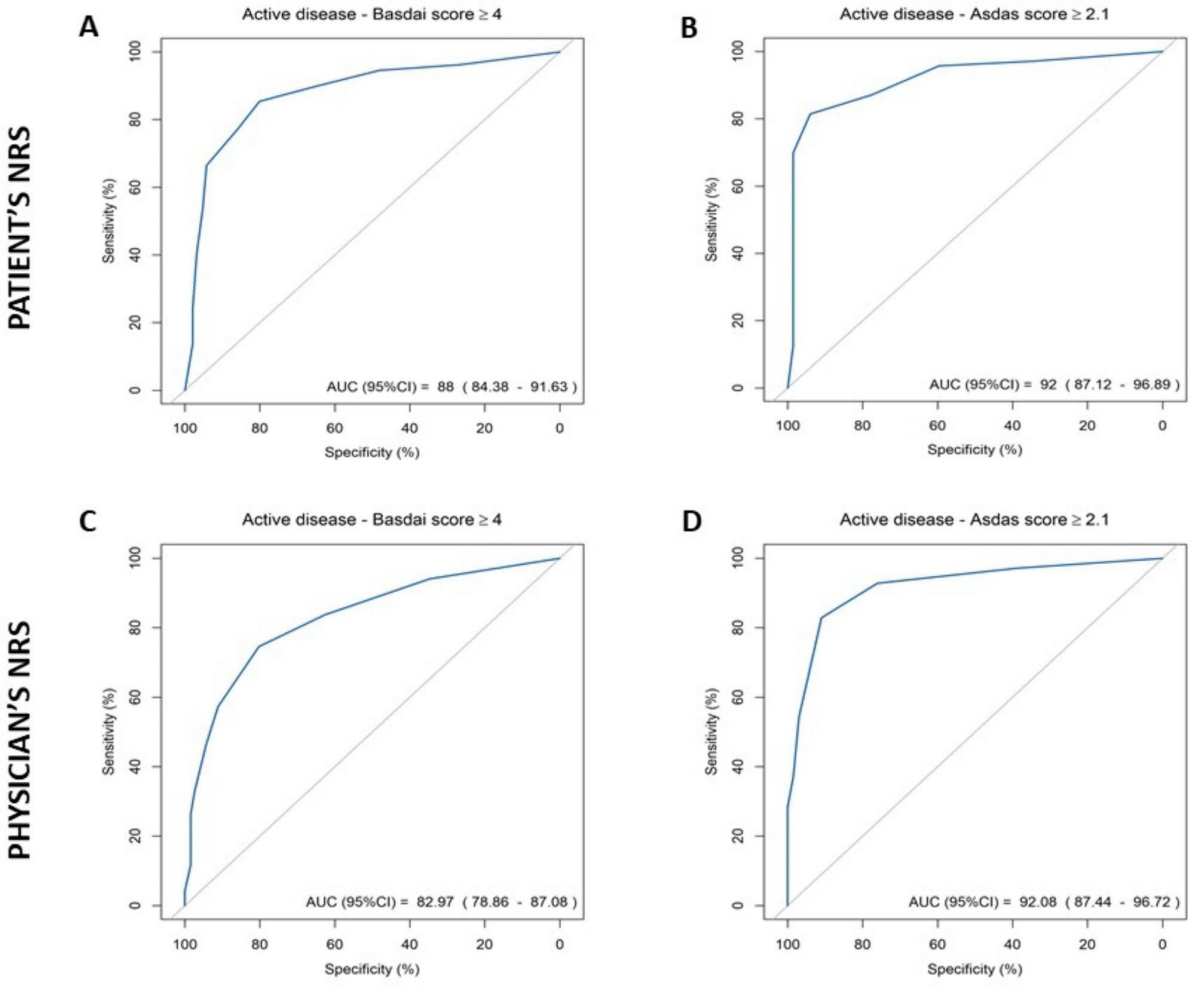
AUC of patient’s (A, B) and physician’s (C, D) NRS for predicting disease activity according to BASDAI and ASDAS-CRP *ASDAS-CRP* Ankylosing Spondylitis Disease Activity Score, *AUC* area under the curve, *BASDAI* Bath Ankylosing Spondylitis Disease Activity Index, *CI* confidence interval, *NRS* numeric rating scale

**Table 3:** Perception of disease activity

	All patients	Using only NSAID (n = 42)	Using only TNFi (n = 281)	Using both TNFi and NSAID (n = 55)
**NRS – patient**	4.24 (3.25)	6.67 (2.76)	3.55 (3.11)	5.85 (2.88)
**NRS - physician**	2.85 (2.57)	5.98 (2.47)	2.07 (2.08)	4.44 (2.42)

*Data presented as mean (SD)*

*NRS* numeric rating scale, *NSAID* nonsteroidal anti-inflammatory drug, *SD* standard deviation, *TNFi* tumor necrosis factor inhibitors

### Perception of disease control

Patients perceived their disease to be more active than their physicians. For both patients and physicians, the disease was moderately active (mean NRS scores ≥ 5) for patients treated with TNFi in combination with NSAID or NSAID alone. For patient under TNFi alone, both patients and physicians reported moderate-to-low disease activity; but score mean for disease activity reported was 2.85 for physicians and 4.24 for patients (Table 3).

**Table 4:** Correlation coefficient (95%CI) of patients and physicians’ perceptions of disease activity with the PRO.

	BASDAI	ASDAS-CRP
**NRS (patient)**	0.77 (0.72; 0.80)	0.84 (0.78; 0.88)
**NRS (physician)**	0.68 (0.62; 0.73)	0.81 (0.74; 0.86)

Data presented as Spearman (CI), ρ Spearman test

ASDAS-CRP Ankylosing Spondylitis Disease Activity Sco*re, BASDAI* Bath Ankylosing Spondylitis Disease Activity Index, *CI*, confidence interval, *NRS* numeric rating scale, *NSAID* Nonsteroidal anti-inflammatory drugs, *PRO* patient-reported outcome, *SD* standard deviation, *TNFi* tumor necrosis factor inhibitors

Both patients and physicians’ perceptions were highly correlated to BASDAI and ASDAS-CRP scores (Table 4). Both patients and physicians were able to predict disease activity as active or inactive. All AUC were higher than 0.8 for BASDAI and ASDAS-CRP cut-offs for disease activity (Fig. 1).

## Discussion

This noninterventional and observational study described the disease activity of patients with AS treated with TNFi and/or NSAID for at least 12 weeks in the past 26 weeks in Brazil.

The demographics of the study sample were comparable to previous studies [14, 24–26], as most patients were male and typically had disease onset before the age of 40–45 years. As expected, HLA-B27 presence among patients with AS was high in this study cohort, and these data are in line with previous studies in Latin America [2], although the HLA-B27 frequency was lower than that in studies outside Brazil, which showed as high as 91% frequency of HLA-B27 whereas it was 65% in the current study [24].

Both NSAID and TNFi are efficacious, prescribed for AS control, and the results are as expected from the phase III studies of the evaluated drugs. In the INVISIBLE study, despite having been treated with TNFi and/or NSAID for at least 12 weeks, half of the patients exhibited moderate to high disease activity, whereas ~ 23% showed low activity but still low activity/suboptimal control (not very inactive disease). The majority of patients included in this study was using TNFi alone (mainly adalimumab) and although showing better control of the disease than patients using NSAIDs alone or in combination with TNFi, more than half of the patients using TNFi alone still have not reached adequate disease control (36–38% with active disease plus 27 − 19% with low activity/suboptimal control). A few real-world studies have shown that disease activity scores and CRP decrease among patients with AS after initiating biological therapy [24, 27–29]. However, corroborating with our findings, it has also been reported that 20–40% of AS patients on TNFi showed an inadequate response or become intolerant to the treatment over time [30]. Additionally, other studies showed that TNFi is efficacious in reducing disease activity but still might not lead to good or adequate disease control after second and third of anti-TNF treatment [10, 31, 32].

Overall, groups treated with NSAIDs (alone or in combination with TNFi) had worse disease activity results than TNF alone. Although NSAIDs have been proven to be efficacious in symptom reduction and in reducing inflammatory serum biomarkers, they do not always lead to adequate symptom control [33]. Moreover, a transversal unicentric study (N = 152) [14] reported a significantly higher proportion of patients with AS with low disease activity and inactive disease in patients treated with TNFi than in those treated with NSAIDs. In the current study, most patients under NSAID presented inadequate disease control, worse than those using TNFi. This might suggest that many patients under NSAID might be eligible for switching therapy for biologics, as indicated in disease guidelines.

Although the first line of treatment is NSAIDs in AS, the number of patients using NSAIDs was relatively low in this cohort. As this study did not require patients to be under first-line of therapy, most patients might have switched their NSAID to biologics therapy over time, which would explain the higher number of included cases under biologics. Besides, NSAID group showed higher disease activity than the biologics, which might be reflecting patients under their first line of therapy that are failing NSAID and are now eligible for initiating biologics.

However, regardless the treatment received, the proportion of AS patients with low disease activity or inactive disease in the current study is lower than in previous studies, which showed around 75% of AS patients [34] with low disease activity or inactive disease and around 50% of patients with restricted inactive disease after AS treatment [14]. This study included clinics with a high standard care (e.g., national guidelines are followed, detailed standard operating procedures are develop and followed, etc.) in Brazil, which could not be generalized to the entire country; however, it is reasonable to believe that patients in different settings may have worse disease control. Even though included patients were on assumed great care, many still presented with disease activity, bringing to the attention that there might be very complex mechanisms for patients to be unable to achieve adequate treatment. Some possible explanations for treatment failure may be due to [1] non-compliance and non-adherence by patients - studies have shown that lack of knowledge about the disease and consequences of poor compliance could be the reason for this patient behavior; [2] sporadic and not routine use of PROs by healthcare providers – it has been reported that time constraint, insufficient knowledge and lack of integration of PROs into clinical system, are some of the barriers for the implementation of PROs in the clinical practice [35]; [3] lack of effective communication between healthcare providers and patients [36] - healthcare providers, including physician, improving communication with patients can further improve overall management of the disease [37, 38]; and [4] clinical inertia – failure of physicians to initiate, change or intensify therapy when required especially when there is evidence of disease activity for chronic disease such as AS [39, 40].

AS is a multidimensional inflammatory disease requiring overall management of the disease including morbidities, complications, and disease progression. Adequate care is possible but requires a broad and multi-disciplinary effort. Healthcare providers must strive to get the right and early diagnosis, and also to treat the right patient, at the right time, with the right treatment, at the right dose. Patients should be encouraged to self-management and self-advocacy through effective listening and empathy by healthcare providers. Physicians are essential for that, but pharmacists, physiotherapists, community or family healthcare providers, and many other professionals can help supporting and engaging the correct and adequate AS treatment, pharmacological and non-pharmacological. A suboptimal management of chronic disease such as AS can further increase risk of subsequent adverse health outcomes such as fatigue, pain, impaired function, and psychosocial problems; nevertheless, misdiagnosis and other factors that may lead to undesirable treatment outcome may occur. [41, 42].

For chronic pain disease such as AS, therapeutic decisions and assessment of disease activity rely on PROs in addition to physicians’ clinical evaluation [43] as they are reliable and effective in reflecting changes in disease activity over time [44]. A qualitative study assessed PROs in patients with AS indicated that PROs measures should be routinely used in outpatient settings to help improve shared decision-making discussions between patients and physicians [37]. The patient perspective is critical to make continuous improvement in the treatment of AS by encouraging appropriate treatment switching and escalation. In this study, there was a slight patient–physician discrepancy regarding the perception of AS disease activity. The patients perceived their disease to be more active than the physicians; this is in keeping with data from a systematic review of literature [38, 44]. A plausible explanation could be, patients solely subjective perception of pain and discomfort, so they tend to perceive more severe disease not only due to the disease status but also psychological distress and comorbidities [44].

This study has some limitations. Patients who regularly visited their physicians in clinics were more likely to be approached and enrolled in this study. The investigator selection bias was minimized by enrolling consecutive patients who fulfilled the eligibility criteria. Patients presenting with symptoms were more likely to get a medical consultation and to be included in this study, as they were visiting the clinics. Few CRP were available to allow ASDAS-CRP evaluation, leaving only BASDAI for disease activity determination; and because it relies solely on patient’s perception, it could have inflated disease activity in this study. It is well stablished that a psychological distress is commonly a trigger and an aggravating factor to nociplastic pain; therefore, stress or other problems in patient care caused by the COVID-19 pandemic may influence the results, including the perception of the disease (overall more active to patient than to their physician) and the proportion of AS patients with inactive or low disease activity (lower in this study than in previous ones). Also, race/ethnicity data was not collected, which may limit the understanding of these population [45, 46]. The treatment was retrospectively assessed to reduce physician bias with regard to treatment selection and indication; however, patients and disease characteristics are key points for physician’s choice of treatment, so comparison among treatment groups within this study should be done with caution.

The strength of this study is the nature of it, which reflects real world scenario, where physicians were not biased in assigning treatment, and patient’s outcomes reflects what is happening in clinical practice. The INVISIBLE-BRAZIL study has highlighted the importance of seeking for better disease control, improving monitoring and treatment selection. Moreover, controlling disease manifestation is important to maintain or improve patients’ quality of life.

## Conclusion

In this Brazilian real world study, half of patients with AS treated with TNFi and/or NSAID exhibited active disease or low activity/suboptimal disease control, despite being treated for at least 12 weeks. Results from this study raise the need for a widespread use of disease monitoring and PROs can improve physicians’ understanding of disease activity and aid treatment decision-making, which can further improve patient satisfaction and management of the disease. More studies are needed to understand the factors associated with the inadequate disease control.

## Study limitation

The results of inadequate disease control of the present study are probably impacted by the profile of patients included in the study considering that it is known that individuals with painful sensitization and fibromyalgia that fulfill criteria for spondylarthritis show better treatment outcomes than patients with symptoms without an inflammation biomarker. The treatment duration can also play an important bias in the assessment of disease activity even when in this study the treatment with TNFi, NSAID or combination of both were, at least, equal or superior to 6 months. Other limitation of this study is regarding that it was not assessed pre study treatment for AS as well as the questionnaire obtaining process and timing are potential biases.

## Electronic supplementary material

Below is the link to the electronic supplementary material.

Supplementary Material 1

## Data Availability

The datasets generated during and/or analyzed during the current study are available from the corresponding author on reasonable request.

## Abbreviations

AS: ankylosing spondylitis
ASDAS-CRP: ankylosing spondylitis disease activity score
BASDAI: bath ankylosing spondylitis disease activity index
CRP: C-reactive protein
IQR: interquartile range
NRS: numeric rating scale
NSAIDs: nonsteroidal anti-inflammatory drugs
PRO: , patient-reported outcome
SD: standard deviation
TNFi: tumor necrosis factor inhibitors

## References

[1] Jiang Y, Yang M, Wu H, Song H, Zhan F, Liu S (2015). The relationship between disease activity measured by the BASDAI and psychological status, stressful life events, and sleep quality in ankylosing spondylitis. Clin Rheumatol.

[2] Citera G, Bautista-Molano W, Peláez-Ballestas I, Azevedo VF, Perich RA, Méndez-Rodríguez JA (2021). Prevalence, demographics, and clinical characteristics of Latin American patients with spondyloarthritis. Adv Rheumatol.

[3] Ward MM, Deodhar A, Gensler LS, Dubreuil M, Yu D, Khan MA (2019). 2019 Update of the American College of Rheumatology/Spondylitis Association of America/Spondyloarthritis Research and Treatment Network Recommendations for the Treatment of Ankylosing Spondylitis and Nonradiographic Axial Spondyloarthritis. Arthritis Care Res (Hoboken).

[4] Sieper J, Braun J, Rudwaleit M, Boonen A, Zink A (2002). Ankylosing spondylitis: an overview. Ann Rheum Dis.

[5] Sunkureddi P, Gibson D, Doogan S, Heid J, Benosman S, Park Y (2018). Using Self-Reported Patient Experiences to Understand Patient Burden: Learnings from Digital Patient Communities in Ankylosing Spondylitis. Adv Ther.

[6] van der Heijde D, Ramiro S, Landewé R, Baraliakos X, Van den Bosch F, Sepriano A (2017). 2016 update of the ASAS-EULAR management recommendations for axial spondyloarthritis. Ann Rheum Dis.

[7] Sampaio-Barros PD, Keiserman M, Souza M, Ed MP, Md, Ximenes AC, Azevedo VF (2013). Recomendações sobre diagnóstico e tratamento da espondilite anquilosante. Revista brasileira de reumatologia.

[8] Resende GG, Meirelles EdS, Marques CDL, Chiereghin A, Lyrio AM, Ximenes AC (2020). The Brazilian Society of Rheumatology guidelines for axial spondyloarthritis – 2019. Adv Rheumatol.

[9] Strand V, Deodhar A, Alten R, Sullivan E, Blackburn S, Tian H, Gandhi KK, Jugl SM, Conaghan PG. (2021). Pain and Fatigue in Patients With Ankylosing Spondylitis Treated With Tumor Necrosis Factor Inhibitors: Multinational Real-World Findings. J Clin rheumatology: practical Rep rheumatic Musculoskelet Dis, 27(8), doi:10.1097/RHU.0000000000001544. 10.1097/RHU.0000000000001544 PMC8612885 32826654

[10] Corbett M, Soares M, Jhuti G, Rice S, Spackman E, Sideris E, Moe-Byrne T, Fox D, Marzo-Ortega H, Kay L, Woolacott N, Palmer S. (2016). Tumour necrosis factor-α inhibitors for ankylosing spondylitis and non-radiographic axial spondyloarthritis: a systematic review and economic evaluation. Health Technol Assess (Winchester Eng), 20(9), doi:10.3310/hta20090. 10.3310/hta20090 PMC4781282 26847392

[11] van der Heijde D, Schiff MH, Sieper J, Kivitz AJ, Wong RL, Kupper H (2009). Adalimumab effectiveness for the treatment of ankylosing spondylitis is maintained for up to 2 years: long-term results from the ATLAS trial. Ann Rheum Dis.

[12] Braun J, Deodhar A, Inman RD, van der Heijde D, Mack M, Xu S (2012). Golimumab administered subcutaneously every 4 weeks in ankylosing spondylitis: 104-week results of the GO-RAISE study. Ann Rheum Dis.

[13] Burgos-Vargas R, Wei JC-C, Rahman MU, Akkoc N, Haq SA, Hammoudeh M (2016). The prevalence and clinical characteristics of nonradiographic axial spondyloarthritis among patients with inflammatory back pain in rheumatology practices: a multinational, multicenter study. Arthritis Res Therapy.

[14] Moreno M, Arévalo M, Zamora M, Pontes C, Oliva JC, Gratacós J (2021). Comparison of disease activity in patients with ankylosing spondylitis under TNFi or NSAID treatment, is there any difference? An observational study. Reumatología Clínica.

[15] Garrett S, Jenkinson T, Kennedy LG, Whitelock H, Gaisford P, Calin A (1994). A new approach to defining disease status in ankylosing spondylitis: the Bath Ankylosing Spondylitis Disease Activity Index. J Rheumatol.

[16] Machado P, Landewé R, Lie E, Kvien TK, Braun J, Baker D (2011). Ankylosing Spondylitis Disease Activity Score (ASDAS): defining cut-off values for disease activity states and improvement scores. Ann Rheum Dis.

[17] Auleley GR, Benbouazza K, Spoorenberg A, Collantes E, Hajjaj-Hassouni N, van der Heijde D (2002). Evaluation of the smallest detectable difference in outcome or process variables in ankylosing spondylitis. Arthritis Rheum.

[18] Kiltz U, Baraliakos X, Karakostas P, Igelmann M, Kalthoff L, Klink C (2012). The degree of spinal inflammation is similar in patients with axial spondyloarthritis who report high or low levels of disease activity: a cohort study. Ann Rheum Dis.

[19] Kaffel D, bouden s, Maatallah K, Abaza N, Riahi H, Hamdi W, et al. Ab0715 Assessment of Body Composition in Lean Mass and Fat Mass in Spondyloarthritis. Abstracts accepted for Publication2019. p. 1820–1: 10.1136/annrheumdis-2019-eular.6442.

[20] Chen H, Pan T, Chen Y, Chen Y, Huang W, Hsieh T, Lai K, Hung WT, Chou Y, Tseng C, Wu Y, Hsieh C, Lin C (2019). AB0716 establishment of basdai cut-offs for the disease activity states based on asdas cut-offs in taiwanese ankylosing spondylitis patients. Ann Rheum Dis.

[21] Molnar C, Scherer A, Baraliakos X, de Hooge M, Micheroli R, Exer P (2018). TNF blockers inhibit spinal radiographic progression in ankylosing spondylitis by reducing disease activity: results from the Swiss Clinical Quality Management cohort. Ann Rheum Dis.

[22] Cohen J (1988). Statistical Power Analysis for the Behavioral Sciences.

[23] Nascimento TL, Vasconcelos SP, Saturnino LT, Correia MG. Prevalencia da Espondilite Anquilosante: Uma Revisao Sistematica. Value in Health. 2015;18(7):A877

[24] Nossent JC, Sagen-Johnsen S, Bakland G (2019). Disease Activity and Patient-Reported Health Measures in Relation to Cytokine Levels in Ankylosing Spondylitis. Rheumatol Ther.

[25] Arends S, Brouwer E, van der Veer E, Groen H, Leijsma MK, Houtman PM (2011). Baseline predictors of response and discontinuation of tumor necrosis factor-alpha blocking therapy in ankylosing spondylitis: a prospective longitudinal observational cohort study. Arthritis Res Therapy.

[26] Glintborg B, Østergaard M, Krogh NS, Dreyer L, Kristensen HL, Hetland ML (2010). Predictors of treatment response and drug continuation in 842 patients with ankylosing spondylitis treated with anti-tumour necrosis factor: results from 8 years’ surveillance in the Danish nationwide DANBIO registry. Ann Rheum Dis.

[27] de Machado MAdÁ, Almeida AM, Kakehasi AM, Acurcio FdA (2016). Real Life Experience of First Course of Anti-TNF Treatment in Ankylosing Spondylitis Patients in Brazil. Rheumatol therapy.

[28] Lie E, Fagerli KM, Mikkelsen K, Rødevand E, Lexberg A, Kalstad S (2014). First-time prescriptions of biological disease-modifying antirheumatic drugs in rheumatoid arthritis, psoriatic arthritis and axial spondyloarthritis 2002–2011: data from the NOR-DMARD register. Ann Rheum Dis.

[29] Mease PJ, van der Heijde D, Karki C, Liu M, Park Y, Greenberg JD (2018). Tumor Necrosis Factor Inhibitor Discontinuation in Patients with Ankylosing Spondylitis: An Observational Study From the US-Based Corrona Registry. Rheumatol Ther.

[30] Braun J, van den Berg R, Baraliakos X, Boehm H, Burgos-Vargas R, Collantes-Estevez E (2011). 2010 update of the ASAS/EULAR recommendations for the management of ankylosing spondylitis. Ann Rheum Dis.

[31] van der Heijde D, Landewé R, Einstein S, Ory P, Vosse D, Ni L (2008). Radiographic progression of ankylosing spondylitis after up to two years of treatment with etanercept. Arthritis Rheum.

[32] van der Heijde D, Landewé R, Baraliakos X, Houben H, van Tubergen A, Williamson P (2008). Radiographic findings following two years of infliximab therapy in patients with ankylosing spondylitis. Arthritis Rheum.

[33] van der Heijde D, Baraf HS, Ramos-Remus C, Calin A, Weaver AL, Schiff M (2005). Evaluation of the efficacy of etoricoxib in ankylosing spondylitis: results of a fifty-two-week, randomized, controlled study. Arthritis Rheum.

[34] Monti S, Todoerti M, Codullo V, Favalli EG, Biggioggero M, Becciolini A (2018). Prevalence of Ankylosing Spondylitis Disease Activity Score (ASDAS) inactive disease in a cohort of patients treated with TNF-alpha inhibitors. Mod Rheumatol.

[35] Turner GM, Litchfield I, Finnikin S, Aiyegbusi OL, Calvert M (2020). General practitioners’ views on use of patient reported outcome measures in primary care: a cross-sectional survey and qualitative study. BMC Fam Pract.

[36] Azevedo S, Guimarães F, Almeida D, Faria D, Silva J, Rodrigues J, et al. FRI0307 DETERMINANTS OF PATIENT-PHYSICIAN DISCORDANCE IN GLOBAL ASSESSMENT IN SPONDYLOARTHRITIS. 2020;79(Suppl 1): 743.1–744. DOI:10.1136/annrheumdis-2020-eular.3341.

[37] Chakravarty SD, Abell J, Leone-Perkins M, Orbai A-M (2021). A Novel Qualitative Study Assessing Patient-Reported Outcome Measures Among People Living with Psoriatic Arthritis or Ankylosing Spondylitis. Rheumatol Therapy.

[38] Pablos JL, Juanola X, Barbazán C, García Vivar ML, Valenciano AC, Rodriguez-Lozano C, POS0985 DISEASE CONTROL PERCEPTION BY PHYSICIANS AND PATIENTS WITH ANKYLOSING SPONDYLITIS AND PSORIATIC ARTHRITIS IN REAL CLINICAL PRACTICE IN SPAIN (2021). MIDAS STUDY RESULTS.

[39] Aujoulat I, Jacquemin P, Rietzschel E, Scheen A, Tréfois P, Wens J (2014). Factors associated with clinical inertia: an integrative review. Adv Med Educ Pract.

[40] Farup PG, Blix I, Førre S, Johnsen G, Lange O, Johannessen R (2011). What causes treatment failure - the patient, primary care, secondary care or inadequate interaction in the health services?. BMC Health Serv Res.

[41] Clarke A, James S, Ahuja S (2010). Ankylosing spondylitis: inadvertent application of a rigid collar after cervical fracture, leading to neurological complications and death. Acta Orthop Belg.

[42] Yahya F, Gaffney K, Sengupta R (2019). Exploring sub-optimal response to tumour necrosis factor inhibitors in axial spondyloarthritis. Rheumatol Adv Pract.

[43] Eder L, Thavaneswaran A, Chandran V, Cook R, Gladman DD (2015). Factors explaining the discrepancy between physician and patient global assessment of joint and skin disease activity in psoriatic arthritis patients. Arthritis Care Res (Hoboken).

[44] Sacristán JA, Dilla T, Díaz-Cerezo S, Gabás-Rivera C, Aceituno S, Lizán L (2020). Patient-physician discrepancy in the perception of immune-mediated inflammatory diseases: rheumatoid arthritis, psoriatic arthritis and psoriasis. A qualitative systematic review of the literature. PLoS ONE.

[45] Fitzcharles MA, Cohen SP, Clauw DJ, Littlejohn G, Usui C, Häuser W (2021). Nociplastic pain: towards an understanding of prevalent pain conditions. Lancet.

[46] Nicholas M, Vlaeyen JWS, Rief W, Barke A, Aziz Q, Benoliel R (2019). The IASP classification of chronic pain for ICD-11: chronic primary pain. Pain.

