# Supplementary material for: Maintained activity in ankylosing spondylitis patients treated with TNFi and/or NSAID for at least 12 weeks: a cross-sectional study in Brazil
Source: Adv Rheumatol. 2022 Oct 28;62(1):38. doi: 10.1186/s42358-022-00270-3 (PMC9614733; doi:10.1186/s42358-022-00270-3)
Supplement: Supplementary file 1 — Supplementary Material 1 [file 42358_2022_270_MOESM1_ESM.docx]

**Supplementary table 1.** TNFi treatment characteristics of the AS patients included in the study

|  | **Patients on**  **treatment**  n (%) | **Time since diagnosis (y)**  Mean (SD) | **Treatment duration (y)**  Mean (SD) | **Dose (mg)/week**  Median (IQR) | **Main frequency of use (every *x* week)**  n (%) |
| --- | --- | --- | --- | --- | --- |
| ***TNFi monotherapy (n=281)*** | | | | | |
| **Adalimumab** (n=126, 45%) |  |  |  |  | *Every 2w* |
| Active disease | 49 (38.9) | 6.99 (7.00) | 3.87 (2.9) | 20 (20–20) | 47 (95.92) |
| Low or inactive disease | 77 (61.1) | 7.99 (6.68) | 5.05 (3.9) | 20 (20–20) | 66 (85.71) |
| **Certolizumab**  (n=15, 5%) |  |  |  |  | *Every 2w* |
| Active disease | 7 (46.7) | 7.13 (6.51) | 1.62 (1.4) | 100 (100–100) | 5 (71.43) |
| Low or inactive disease | 8 (53.3) | 9.16 (12.83) | 1.15 (1.0) | 100 (100–100) | 4 (50.00) |
| **Etanercept**  (n=54, 19%) |  |  |  |  | *Every 1w* |
| Active disease | 21 (38.9) | 9.52 (9.15) | 4.55 (3.4) | 50 (50–50) | 18 (85.71) |
| Low or inactive disease | 33 (61.1) | 9.57 (5.71) | 5.53 (3.6) | 50 (25–50) | 23 (69.70) |
| **Golimumab**  (n=38, 16%) |  |  |  |  | *Every 4w* |
| Active disease | 16 (42.1) | 5.94 (5.81) | 2.36 (2.5) | 12.5 (12.5–12.5) | 13 (81.25) |
| Low or inactive disease | 22 (57.9) | 7.37 (7.66) | 2.28 (2.6) | 12.5 (12.5–12.5) | 20 (95.24) |
| **Infliximab**  (n=48, 17%) |  |  |  |  | *Every 8w* |
| Active disease | 26 (54.2) | 7.96 (6.74) | 4.38 (4.2) | 50 (37.5–53.1) | 25 (96.15) |
| Low or inactive disease | 22 (45.8) | 11.04 (9.84) | 6.03 (4.1) | 57.7 (50–62.5) | 19 (86.36) |
| **TNFi in combination with NSAID**  (n=55) |  |  |  |  |  |
| Active disease | 46 (83.6) | 7.11 (8.62) | 1.34 (1.8) | - | - |
| Low or inactive disease | 9 (16.4) | 8.01 (8.29) | 1.92 (2.1) | - | - |

*AS* ankylosing spondylitis, *IQR* interquartile range, *NSAID* nonsteroidal anti-inflammatory drug, *SD* standard deviation, *TNFi* tumor necrosis factor inhibitors, *w* weeks, *y* years.

**Supplementary table 2.** NSAID treatment characteristics of patients with AS included in the study

|  | **Patients on**  **treatment**  n (%) | **Time since diagnosis (y)**  Mean (SD) | **Treatment duration (y)**  Mean (SD) | **Dose (mg)/week**  Median (IQR) | **On demand use**  n (%) |
| --- | --- | --- | --- | --- | --- |
| **Naproxen**  (n=10, 24%) |  |  |  |  |  |
| Active disease | 9 (90) | 2.60 (1.14) | 0.91 (0.28) | 500 (500–500) | 2 (22.2) |
| Low or inactive disease | 1 (10) | 6.69 (NA) | 1.19 (NA) | 1000 (NA) | 0 (0) |
| **Celecoxib**  (n=7, 17%) |  |  |  |  |  |
| Active disease | 5 (71.4) | 4.90 (9.27) | 0.53 (0.3) | 200 (200–200) | 1 (16.7) |
| Low or inactive disease | 2 (28.6) | 6.22 (0.53) | 2.27 (0.6) | 200 (200–200) | 2 (100) |
| **Etoricoxib**  (n=6, 14**%)** |  |  |  |  |  |
| Active disease | 6 (100) | 6.48 (5.45) | 3.99 (0.7) | 90 (90–90) | 2 (33.3) |
| Low or inactive disease | 0 | NA | NA | NA | NA |
| **Ketoprofen**  (n=2, 5**%)** |  |  |  |  |  |
| Active disease | 1 (50) | 1.26 (NA) | 14.86 (NA) | 200 (NA) | 1 (100) |
| Low or inactive disease | 1 (50) | 5.79 (NA) | 1.12 (NA) | 200 (NA) | 1 (100) |
| **Diclofenac**  (n=6, 14**%)** |  |  |  |  |  |
| Active disease | 6 (100) | 3.64 (4.50) | 3.23 (0.5) | 50 (50–100) | 1 (16.7) |
| Low or inactive disease | 0 | NA | NA | NA | NA |
| **Meloxicam**  (n=1, 2**%)** |  |  |  |  |  |
| Active disease | 0 | NA | NA | NA | NA |
| Low or inactive disease | 1 (100) | 21.46 (NA) | 20.33 (NA) | 15 (NA) | 1 (100) |
| **Other**  (n=5, 12%) |  |  |  |  |  |
| Active disease | 3 (60) | 10.65 (3.37) | 1.83 (2.3) | NA | NA |
| Low or inactive disease | 2 (40) | 4.50 (0.20) | 2.72 (2.7) | NA | NA |
| **≥2 NSAID**  (n=5, 12%) |  |  |  |  |  |
| Active disease | 4 (80) | 0.89 (0.27) | 1.70 (2.1) | NA | NA |
| Low or inactive disease | 1 (20) | 18.43 (NA) | 1.83 (NA) | NA | NA |

*AS* ankylosing spondylitis, *IQR* interquartile range, *NSAID* nonsteroidal anti-inflammatory drug, *SD* standard deviation, *y* years.
